# Early outcomes after robotic arm-assisted bi-unicompartmental knee arthroplasty compared with total knee arthroplasty: a prospective, randomized controlled trial

**DOI:** 10.1302/0301-620X.103B10.BJJ-2020-1919.R2

**Published:** 2021-10-01

**Authors:** Mark J. G. Blyth, Matthew S. Banger, James Doonan, Bryn G. Jones, Angus D. MacLean, Philip J. Rowe

**Affiliations:** 1 Department of Trauma and Orthopaedics, Glasgow Royal Infirmary, Glasgow, UK; 2 Department of Biomedical Engineering, University of Strathclyde, Glasgow, UK

**Keywords:** Randomized controlled trial, Arthroplasty, Knee, Robotic-assisted surgery, Outcomes, Robotic arm, Randomized Controlled Trial, total knee arthroplasty (TKA), Unicompartmental knee arthroplasty (UKA), patient-reported outcome measures (PROMs), clinical outcomes, BMI, osteoarthritis of the knee, analgesics

## Abstract

**Aims:**

The aim of this study was to compare the clinical outcomes of robotic arm-assisted bi-unicompartmental knee arthroplasty (bi-UKA) with conventional mechanically aligned total knee arthroplasty (TKA) during the first six weeks and at one year postoperatively.

**Methods:**

A per protocol analysis of 76 patients, 43 of whom underwent TKA and 34 of whom underwent bi-UKA, was performed from a prospective, single-centre, randomized controlled trial. Diaries kept by the patients recorded pain, function, and the use of analgesics daily throughout the first week and weekly between the second and sixth weeks. Patient-reported outcome measures (PROMs) were compared preoperatively, and at three months and one year postoperatively. Data were also compared longitudinally and a subgroup analysis was conducted, stratified by preoperative PROM status.

**Results:**

Both operations were shown to offer comparable outcomes, with no significant differences between the groups across all timepoints and outcome measures. Both groups also had similarly low rates of complications. Subgroup analysis for preoperative psychological state, activity levels, and BMI showed no difference in outcomes between the two groups.

**Conclusion:**

Robotic arm-assisted, cruciate-sparing bi-UKA offered similar early clinical outcomes and rates of complications to a mechanically aligned TKA, both in the immediate postoperative period and up to one year following surgery. Further work is required to identify which patients with osteoarthritis of the knee will derive benefit from a cruciate-sparing bi-UKA.

Cite this article: *Bone Joint J* 2021;103-B(10):1561–1570.

## Introduction

Cemented total knee arthroplasty (TKA) is a highly successful,^1^ cost-effective,^2^ and commonly performed^3^ operation for patients with osteoarthritis (OA) of the knee. However, postoperative dissatisfaction has frequently been reported.^4^ The function of the knee is often only partially restored following TKA, with poorer kinematics than in age-matched controls.^5-8^ Mahomed et al^9^ reported that 13.1% of 857 patients were somewhat dissatisfied, while 68 patients (7.9%) were very dissatisfied with their ability to participate in recreational activities following TKA.

An accepted shortcoming of most TKAs is the associated resection of one or both cruciate ligaments, which are still functional in most patients who undergo TKA, and may be of critical importance in the younger, more active patients. The importance of the cruciates to natural knee motion has led to the development of several different designs of TKA. Implants designed to include the resection of one or both cruciates feature modifications intended to mimic the function of the removed structures. Bi-cruciate-retaining TKAs have also been developed.^10^ Currently, there is a lack of consensus about the superiority of cruciate-retaining versus cruciate-sacrificing surgery on patient-reported outcomes,^11-13^ despite clear kinematic benefits in retaining these ligaments.^14^

The compartmental approach to TKA aims to deal with only those parts affected by OA by minimizing the resection of bone and cartilage and conserving the ligaments, notably the cruciates.^15^ Unicompartmental knee arthroplasty (UKA) is the most widely adopted example of the compartmental approach, for which similar clinical outcomes have been reported compared with TKA.^16^ Robotic technology has been shown to be beneficial in compartmental knee arthroplasty in a previous randomized controlled trial (RCT) from our institution.^17^ We found less early postoperative pain in 64 patients undergoing robotic arm-assisted UKA compared with 62 conventional manual UKAs at all timepoints up to three months postoperatively.^1^

In patients with more extensive joint involvement, the compartmental approach may include resurfacing of two or even three compartments. In a recent systematic review and meta-analysis, Wada et al^18^ reviewed the sparse literature on this subject. They concluded that there was a need for a methodologically sound comparison of the efficiency of two UKAs as an alternative treatment to TKA in patients with isolated compartmental OA and an intact anterior cruciate ligament (ACL).^18^

The compartmental approach has been performed with manual instrumentation,^19^ computer-assisted navigation,^20^ and more recently with robotic assistance.^11,21^ All combinations of bi-UKA (medial and lateral, medial and patellofemoral, and lateral and patellofemoral) have been described.^22^ A tricompartmental approach has also been described in case reports.^23,24^ Most studies have focused on the combination of medial and patellofemoral compartmental resurfacing; the other combinations have been much less studied.

The bicondylar concept (medial and lateral) is not new, with Gunston^25^ in 1971, Laskin^26^ in 1978, and Goodfellow and O’Connor^19^ in 1986 reporting results using conventional instrumentation. The introduction of robotic arm-assisted technology in the last decade has provided surgeons with a tool which has much higher precision,^27,28^ allowing us to revisit the technique again. We have recently shown that bi-UKA surgery maintains constitutional joint line anatomy better when compared with mechanically aligned TKA.^29^ The aim of the current study was to identify whether this anatomical, bone- and soft-tissue-sparing, kinematic approach, in the same series of patients, led to a shorter length of stay (LOS) in hospital, faster early recovery, and improved outcomes one year postoperatively. The primary outcome measure was the percentage of patients with a bi-phasic (normal) curve during gait (level walking) at this time. As secondary outcomes, we recorded the use of analgesics and measures of standing, walking, stair climbing, pain, stiffness, and satisfaction in the first six postoperative weeks and patient-reported outcome measures (PROMs) at three months and one year following surgery. We also compared the complications between the two groups and the patients’ perception of their treatment as a measure of unblinding.

## Methods

**Fig. 1:**
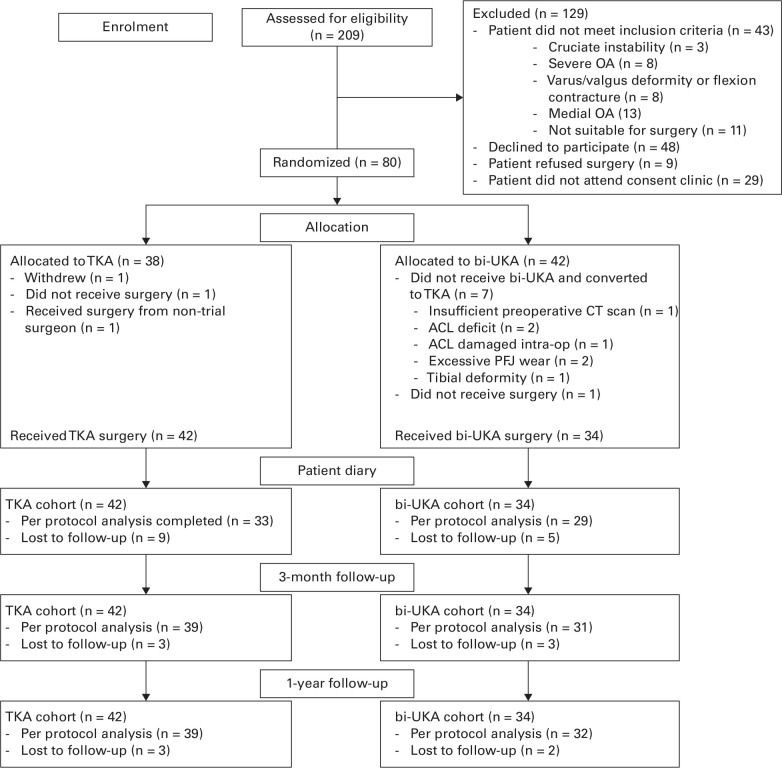
CONSORT diagram showing the flow of patients through the study. bi-UKA, bi-unicompartmental knee arthroplasty; OA, osteoarthritis; TKA, total knee arthroplasty.

Patients on the waiting list for TKA were screened for possible recruitment to the Total versus Robotic assisted bi-UniCompartmental Knee (TRUCK) trial (ISRCTN 12151461). Inclusion and exclusion criteria were confirmed by the treating clinician prior to the patients’ preoperative clinical appointment. Specific exclusion criteria are shown in the CONSORT diagram (Figure 1). Research nurses approached potential participants to obtain consent and collect preoperative data. The trial was a prospective, randomized, single-centre study comparing clinical outcomes in patients undergoing surgery for bi-compartmental OA of the knee, using either conventional manual TKA or robotic arm-assisted bi-UKA.

Patients were eligible for inclusion if they had medial and lateral compartment OA suitable for treatment with a standard unconstrained TKA, with clinically intact cruciate and collateral ligaments and were willing and able to provide informed consent. Exclusion criteria involved patients with rheumatoid or other inflammatory arthropathy, a varus or valgus deformity of > 15°, or a fixed flexion contracture of > 10°, single-compartment OA suitable for an isolated UKA, or radiological evidence of patellofemoral OA > Kellgren and Lawrence grade III.^30^ Patients were not specifically excluded on the location of their symptoms, in particular the patellofemoral joint. Patients were, however, excluded if they had undergone previous surgery to the knee which might affect the outcome of the arthroplasty such as anterior or posterior cruciate ligament reconstruction, except arthroscopy, or those with significant OA in the spine or other lower limb joints which might alter their gait and therefore the primary outcome measure.

The study complied with the principles of the Declaration of Helsinki^31^ and received ethical approval from the West of Scotland Research Ethics Committee (14/WS/0134).^32^ The bi-UKA technique, simultaneously replacing both medial and lateral compartments, was an off-label use of the MAKO System (Stryker, USA) at the time of registration of the trial. Permission for this specific use of the robotic system was obtained via a Clinical Trials Notification (CI/2014/0032) with the Medicines and Healthcare products Regulatory Agency (MHRA). The study was registered with the International Standard Randomized Controlled Trial Number Registry (ISRCTN 12151461).

A total of 209 patients were screened, and 80 were recruited into the trial (Figure 1 and Table I).^32^ Patients were randomized to one of two treatment arms using a web-based system, with all operations sub-randomized to one of three surgeons (MJGB, BGJ, ADM), with extensive experience in TKA, robotic-assisted, and computer-navigated knee surgery.^33,34^ The surgeon and theatre team were aware of the treatment allocation but patients remained blinded. All trial data were collected by a blinded research nurse or research associate at the investigating hospital. Randomization led to similar patient demographics in both groups, with the exception of significantly more patients undergoing bi-UKA using walking aids prior to surgery than those undergoing TKA (Table I). This appears to have been an anomaly as there were no other differences in any of the preoperative PROMs, specifically University of California, Los Angeles (UCLA) Activity Score^35^ and Timed Up and Go Test^36^ (Table I and Table II). Two patients did not undergo surgery following randomization (one TKA and one bi-UKA), one patient had a TKA from a non-trial surgeon and was withdrawn from the study, and one patient withdrew from the trial (one TKA).

**Table I.** Preoperative demographic data for the control (total knee arthroplasty) and intervention (bi-unicompartmental knee arthroplasty) groups.

Variable	TKA	bi-UKA	p-value
Patients, n	42	34	
Mean age, yrs (SD)	70.4 (7.1)	68.7 (7.7)	0.342*
Sex, M:F	21:21	17:17	1.000†
Mean height, m (SD)	1.63 (0.11)	1.62 (0.11)	0.263*
Mean weight, kg (SD)	86.8 (15.7)	83.8 (14.5)	0.408*
Mean BMI, kg/m^2^ (SD)	32.6 (5.5)	32.4 (6.7)	0.869*
Walk with aids, Y:N	8:34	15:19	0.018†
Smoker, Y:N	2:40	3:30	0.455†
Operated knee, R:L	17:25	16:18	0.564†
Median HADS anxiety (IQR)	5 (1 to 10)	5 (2.25 to 9.75)	0.468‡
Median HADS depression (IQR)	5 (3 to 9)	5 (3 to 8)	0.758‡

* Independent-samples *t*-test.

† Chi-squared test.

‡ Mann-Whitney U test.

bi-UKA, bi-unicompartmental knee arthroplasty; HADS, Hospital Anxiety and Depression Scale; IQR, interquartile range; SD, standard deviation; TKA, total knee arthroplasty.

**Table II.** Clinical scores.

Variable	TKA	bi-UKA	p-value*
**Median NKSS knee (IQR)**			
Preoperative	41 (37.5 to 47.5)	42 (36.5 to 50.0)	0.602*
3 mths	48 (41.5 to 53.0)	46 (38 to 52)	0.838*
1 yr	51.5 (42 to 56)	48 (43.0 to 55.5)	0.635*
**Median NKSS function (IQR)**			
Preoperative	52 (40.0 to 73.5)	56 (44.75 to 80.5)	0.379*
3 mths	102 (80.5 to 120.0)	90 (67 to 115)	0.318*
1 yr	109.5 (82.5 to 137.5)	102.5 (77.0 to 120.8)	0.085*
**Median NKSS total (IQR**)			
Preoperative	99 (81.0 to 118.5)	102 (81.2 to 124.3)	0.369*
3 mths	148 (122.0 to 170.5)	140 (115 to 166)	0.384*
1 yr	159 (127.5 to 192.8)	156 (119.3 to 167.3)	0.122*
**Median OKS (IQR)**			
Preoperative	18.5 (14.00 to 26.75)	19 (14.0 to 25.5)	0.751
3 mths	35 (27 to 42)	34 (25 to 39)	0.523
1 yr	37 (29 to 45)	39 (30.5 to 43.0)	0.970
**Mean pain VAS (SD)**			
Preoperative	7.0 (2.0)	6.6 (2.2)	0.744
3 mths	2.1 (2.0)	2.4 (2.4)	0.932
1 yr	1.9 (1.9)	1.6 (2.0)	0.443
**Mean stiffness VAS (SD)**			
Preoperative	6.9 (2.1)	6.6 (2.0)	0.374
3 mths	3.2 (2.4)	3.1 (2.2)	0.938
1 yr	2.4 (2.3)	2.1 (2.3)	0.357
**Median FJS (IQR)**			
Preoperative	N/A	N/A	N/A
3 mths	16 (5.5 to 24.0)	12 (4 to 25)	0.961
1 yr	20.5 (10.25 to 28.75)	19.0 (14.25 to 31.00)	0.525
**Mean EQ-5D-3L VAS (SD)**			
Preoperative	69.8 (16.91)	72.2 (15.3)	0.491
3 mths	82.5 (12.56)	78.0 (21.18)	0.768
1 yr	78.9 (15.7)	74.9 (22.3)	0.723
**Mean EQ-5D-3L index (SD)**			
Preoperative	0.45 (0.31)	0.44 (0.30)	0.896
3 mths	0.71 (0.27)	0.68 (0.3)	0.395
1 yr	0.76 (0.21)	0.73 (0.32)	0.638
**Mean ROM, ° (SD)**			
Preoperative	95.6 (23.3)	101.1 (18.7)	0.407*
3 mths	96.0 (20.0)	95.2 (14.21)	0.392*
1 yr	110.7 (9.4)	107.1 (12.9)	0.369*
**Median UCLA activity scale (IQR)**			
Preoperative	3 (3 to 4)	3 (3 to 5.25)	0.359
3 mths	4 (3 to 6)	4 (3 to 6)	0.796
1 yr	5 (4 to 6)	5.5 (3 to 6)	0.734
**Satisfied, Y:N**			
Preoperative	N/A	N/A	N/A
3 mths	32:9	23:8	0.702‡
1 yr	32: 9	28:4	0.295‡
**Mean Timed Up and Go, secs (SD)**			
Preoperative	14.2 (8.3)	14.1 (6.2)	0.665
3 mths	10.5 (2.8)	13.2 (11.6)	0.550
1 yr	11.2 (5.9)	10.9 (5.6)	0.914
**Mean Stair Climb Test, secs (SD)**			
Preoperative	28.0 (14.2)	26.0 (13.2)	0.667
3 mths	24.2 (12.3)	22.5 (8.2)	0.805
1 yr	19.8 (9.1)	17.1 (6.6)	0.271
**Mean quadricep strength, Nm (SD)**			
Preoperative	71.1 (37.8)	78.5 (33.0)	0.261
3 mths	N/A	N/A	N/A
1 yr	91.5 (39.6)	85.6 (32.1)	0.568

* Independent-samples *t*-test.

† Mann-Whitney U test.

‡ Chi-squared test.

bi-UKA, bi-unicompartmental knee arthroplasty; EQ-5D-3L, EuroQol five-dimension three-level questionnaire; FJS, Forgotten Joint Score; IQR, interquartile range; N/A, not available; NKSS, New Knee Society Score; Nm, Newton metres; OKS, Oxford Knee Score; ROM, range of motion; SD, standard deviation; TKA, total knee arthroplasty; UCLA, University of California, Los Angeles; VAS, visual analogue scale.

**Fig. 2:**
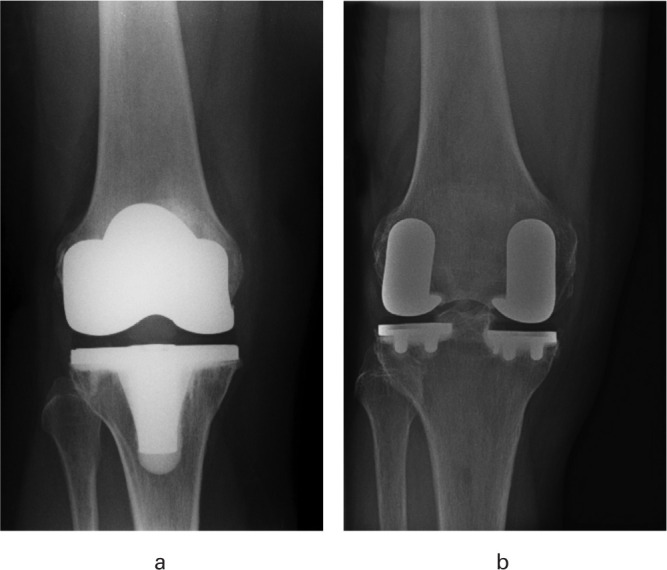
Anteroposterior radiographs of a) total knee arthroplasty using a NexGen LPS-Flex, and b) bi-unicompartmental knee arthroplasty using the medial and lateral Restoris compartmental implants.

Patients were either randomized to the control (TKA) or intervention (bi-UKA) group at a 1:1 ratio. Those randomized to a TKA were treated with a NexGen LPS implant (Zimmer, USA), a fixed-bearing bi-cruciate-sacrificing total condylar TKA using traditional instrumentation without patellar resurfacing (Figure 2a). The aim was to achieve a neutral hip-knee-ankle axis (HKAA) with both femoral and tibial components implanted perpendicular to the mechanical axis.

Those allocated to a bi-UKA were treated with a medial and lateral Restoris MCK (MultiCompartmental Knee) fixed-bearing onlay implant, performed with the aid of the MAKO Robotic-Arm Assisted Technology (Figure 2b) (Stryker, USA). The aim was to resurface the medial and lateral compartments, reconstructing each patient’s constitutional alignment by manually re-tensioning the collateral ligament on the more affected side of the joint (medial collateral ligament for varus OA; lateral collateral ligament for valgus OA). The less affected side was then resurfaced without requiring specific ligament rebalancing. Neither the trochlea nor patella was resurfaced, nor was there a specific need to remove patellar osteophytes or remove overhanging lateral facets. Circumferential denervation of the patella was not performed. The Outerbridge classification^37^ of the degenerative changes in each compartment were recorded during surgery.

An identical medial parapatellar incision and approach was used in both groups throughout. The pins used for the navigation arrays in the bi-UKA group were incorporated within the initial incision.

The patients kept a diary to record their progress daily for the first week and weekly for the next five weeks. The diaries contained Pain and Stiffness visual analogue scores (VAS) and questions about walking, sitting, stair climbing, and household tasks, which have been used in a previous study at our institution.^17^ They attended clinic appointments preoperatively, and at three months and one year following surgery, and research staff performed physical assessments and assisted them when completing PROM questionnaires, including the Oxford Knee Score (OKS),^38^ New Knee Society Score (NKSS),^39^ Forgotten Joint Score (FJS),^40^ EuroQol five-level three-dimension questionnaire (EQ-5D-3L),^38^ UCLA activity score,^35^ Hospital Anxiety and Depression Scale (HADS),^41^ pain and stiffness VAS scores, satisfaction, range of motion (ROM), quadriceps strength, Timed Up and Go (TUG), and stair climbing test.^36^ Complications were recorded at each visit and a test of the blinding exercise was conducted at one year to assess the potential for bias from unintentional unblinding that might have occurred. The maximal isometric quadriceps strength (torque) was calculated from the mean of three measurements using a fixed myometer (MIE; Medical Research, UK). This was normalized by the moment arm (knee to strap) to calculate the torque. The TUG test recorded the time taken for the patient to rise from a chair, walk three metres, turn around, walk back to the chair, and sit down again. Similarly, the stair climbing test was recorded as the time taken to ascend, turn, and descend a 20-step flight of stairs. These assessments were made by a research member of staff (see Acknowledgements) and were performed in a controlled environment.

### Statistical analysis

The primary outcome measure was the percentage of patients with a bi-phasic (normal) curve during gait (level walking) at one year following surgery. In order to detect a significant difference in bi-phasic gait between the two groups at this time, 40 patients per group (including 10% loss to follow-up) were required.

A post-hoc power calculation using G*Power v. 3.1^42^ was performed on the secondary outcome measures described above and at 80% power and an α of 0.05. In order to determine a minimally important clinical difference (MICD) of five points on the OKS, 60 patients per group were required. Using these criteria, the power calculations suggested that a sample size of 80 patients would be powered at 57% for the OKS.

The results were analyzed using intention to treat and per protocol approaches. No differences were observed between these approaches. The results of the per protocol analysis are presented. This approach allowed the inclusion of seven patients who were randomized but were not treated with a bi-UKA to be included in the TKA group. The reasons for these crossovers include: one patient had a low resolution preoperative CT scan that prevented surgical planning, two were found to have excessive patellofemoral joint (PFJ) OA intraoperatively, one had a tibial deformity, one had their ACL damaged intraoperatively, and two were found to be ACL deficient at the time of surgery (one of the contraindications of the bi-UKA technique).

Analysis of differences between the two groups was undertaken using either paired *t*-tests or Mann-Whitney U tests for parametric or non-parametric data to compare variables and PROMs at each timepoint, respectively. When appropriate, categorical data were analyzed using chi-squared tests using statistical software (GraphPad Prism v. 6; GraphPad, USA). Multiple imputation models addressed missing data in the clinical outcomes, and this was further analyzed using mixed models linear regression analysis to compare PROMs between interventions at all timepoints (SPSS v. 24; IBM, USA). A p-value < 0.05 was considered significant.

## Results

There was a significant increase in mean operating time in the bi-UKA group (TKA 96.8 minutes (standard deviation (SD) 15.8) and bi-UKA 159.4 minutes (SD 20.1); p < 0.001, independent-samples *t*-test) although the median LOS was unaffected (TKA three days (interquartile range (IQR) 2.75 to 5.25) and bi-UKA three days (IQR 2 to 4); p = 0.156, Mann-Whitney U test). The increase in operating time in the bi-UKA group was not affected by a learning curve for the surgeons as the operating time did not improve during the study.

**Fig. 3:**
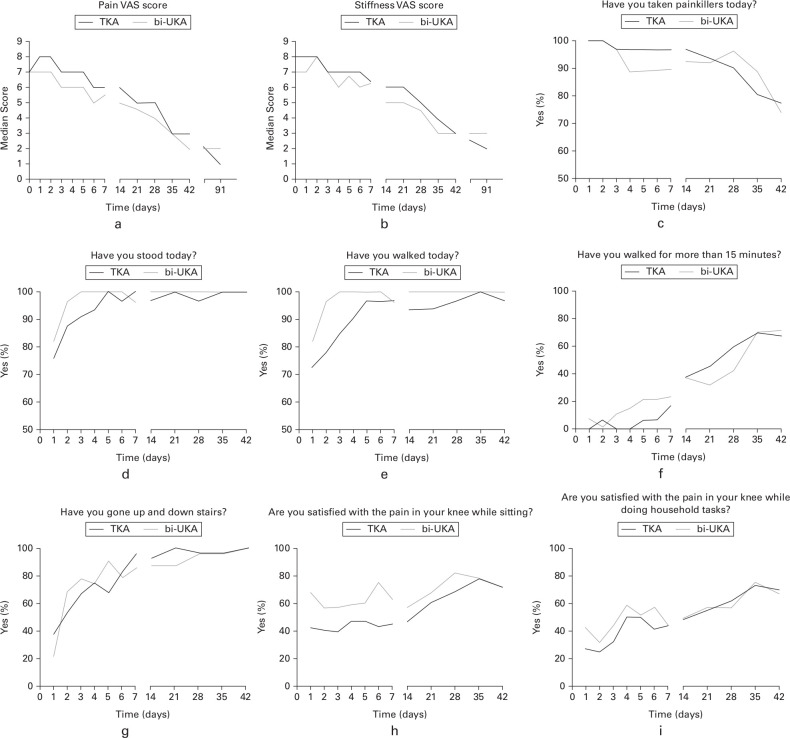
The patients' diaries completed during recovery following total knee arthroplasty (TKA) and bi-unicompartmental knee arthroplasty (UKA). Median a) pain and b) stiffness visual analogue scale (VAS) scores preoperatively, in recovery period (weeks 0 to 6), and at three months. The responses (c to i) are presented as the percentage of patients responding in the positive to each question.

The results from the patients’ diaries during the first six postoperative weeks are summarized in Figure 3. Despite some differences at various timepoints, there were no significant differences between the groups during this time. The VAS scores for pain and stiffness were similar in the two groups. During the first week patients in the bi-UKA group took fewer painkillers, were able to stand, walk, walk for longer, and were more satisfied with their knee while seated than those in the TKA group. However, this trend did not reach statistical significance. In particular, five patients (15.1%) and three patients (9.6%) of the TKA group reported being unable to walk on days 3 and 4 following surgery, while all those in the bi-UKA group were able to walk at these times (p = 0.056 and p = 0.241, respectively, Fisher’s exact test). However, both groups reported similar levels of satisfaction in their knee while doing household tasks and climbing stairs.

There was no significant difference between the groups for any of the PROMS which were analyzed at three months and one year postoperatively (Table II). Additionally, when each PROM was analyzed longitudinally, although there were significant improvements in both groups, there were no significant differences between the groups.

Sub-group analysis based on preoperative scores showed no differences in outcome between the groups in patients who were more active prior to surgery (UCLA Activity Score of < 4 vs ≥ 4), had less anxiety or depression (HADS of < 8 vs ≥ 8), or were overweight (BMI of < 35 kg/m^2^ vs ≥ 35 kg/m^2^). Although trends were seen in some subgroups, there was no clear advantage in either type of operation (data not shown). Importantly, as the trochlea is not resurfaced in a bi-UKA, an analysis was carried out based on the state of the PFJ using the Outerbridge classification. This showed no significant differences in the preoperative distribution of severe PFJ OA between the two groups. Neither was there a difference in the outcomes between the two groups at any time in patients with mild (Outerbridge 0 to 3) or with more severe PFJ OA (Outerbridge 3 or 4) (Supplementary Material).

The complications were reported as those which occurred within three months or between three months and one year following surgery. This separates those relating to the surgery from those arising at a longer period of time postoperatively. There was no significant difference between the rate of complications in the two groups at these two periods of time (Table III). These rates will continue to be monitored.

**Table III.** Categorization and frequency of complications.

Complication, n (%)	< three months postoperatively		> three months postoperatively		Cumulative		p-value*
	TKA	bi-UKA	TKA	bi-UKA	TKA	bi-UKA	
Total	13 (31.0)	10 (29.4)	9 (21.4)	9 (26.5)	22 (52.4)	19 (55.9)	0.760
Wound leakage	2 (4.8)	0 (0.0)	2 (4.8)	0 (0.0)	4 (9.5)	0 (0.0)	0.110
Upper GI complaints	2 (4.8)	0 (0.0)	2 (4.8)	0 (0.0)	4 (9.5)	0 (0.0)	0.110
Cellulitis	0 (0.0)	2 (5.9)	0 (0.0)	1 (2.9)	0 (0.0)	3 (8.8)	0.090
Revision surgery	1 (2.4)	1 (2.9)	0 (0.0)	0 (0.0)	1 (2.4)	1 (2.9)	1.000
CVA	1 (2.4)	1 (2.9)	0 (0.0)	0 (0.0)	1 (2.4)	1 (2.9)	1.000
MUA	0 (0.0)	1 (2.9)	0 (0.0)	1 (2.9)	0 (0.0)	2 (5.9)	0.208
Readmission	0 (0.0)	1 (2.9)	0 (0.0)	1 (2.9)	0 (0.0)	2 (5.9)	0.208
Proven superficial infection	1 (2.4)	1 (2.9)	0 (0.0)	0 (0.0)	1 (2.4)	1 (2.9)	1.000
Postop review	1 (2.4)	1 (2.9)	0 (0.0)	1 (2.9)	1 (2.4)	2 (5.9)	0.588
Fall	1 (2.4)	1 (2.9)	1 (2.4)	2 (5.9)	2 (4.8)	3 (8.8)	0.648
Postop persistent pain	1 (2.4)	1 (2.9)	1 (2.4)	3 (8.8)	2 (4.8)	4 (11.8)	0.389
Unblinding	0 (0.0)	0 (0.0)	1 (2.4)	0 (0.0)	1 (2.4)	0 (0.0)	1.000
Skin problems	1 (2.4)	0 (0.0)	1 (2.4)	0 (0.0)	2 (4.8)	0 (0.0)	0.490
Extended hospitalization	1 (2.4)	0 (0.0)	0 (0.0)	0 (0.0)	1 (2.4)	0 (0.0)	1.000
Respiratory infections	1 (2.4)	0 (0.0)	1 (2.4)	0 (0.0)	2 (4.8)	0 (0.0)	0.490

* Chi-squared test.

bi-UKA, bi-unicompartmental knee arthroplasty; CVA, cardiovascular arrest; GI, gastrointestinal; MUA, manipulation under anaesthesia; TKA, total knee arthroplasty.

In order to determine whether blinding was maintained during follow-up, the patients were asked, at one year following surgery, “Do you believe you know which surgery you received?” and then, “If yes, which treatment did you receive?”. This showed that 33 patients remained unsure of their allocation (20 TKA/13 bi-UKA), while 31 believed they knew which procedure they had received (Table IV). Interestingly, 25 of the 31 “unblinded” patients believed that they had undergone bi-UKA surgery despite their actual allocation (ten TKA/15 bi-UKA; 60% correct). The remaining six “unblinded” patients believed they had received a TKA (five TKA/one bi-UKA; 83% correct). Patients were not asked how they had become “unblinded”; however, on the basis of this failure to guess correctly, we believe that there is no evidence that true unblinding occurred.

**Table IV.** Comparison of clinical scores based on the patients' perception of the allocation of treatment and those who were unsure of their allocation.

Variable	TKA	bi-UKA	Unsure
**Total, n**			
TKA	5	10	20
Bi-UKA	1	15	13
Correct, %	83	60	
**Mean pain VAS (SD)**			
Preop	7.4 (1.8)	6.4 (2.7)	7.0 (1.8)
1 yr	4.0 (3.0)	1.1 (1.1)*	1.9 (2.6)†
**Mean OKS (SD)**			
Preop	16.7 (10.1)	22.2 (9.3)	19.4 (7.6)
1 yr	31.3 (11.1)	38.8 (8.3)	37.0 (9.5)
**Mean NKSS (SD)**			
Preop	94.0 (21.4)	103.4 (28.4)	100.1 (27.0)
1 yr	137.1 (39.0)	155.4 (31.4)	155.2 (38.4)

* Perceived treatment TKA vs perceived treatment bi-UKA; p = 0.002, one-way analysis of variance with Fisher’s least significant difference post-test for multiple comparisons.

† Perceived treatment TKA vs unsure participants; p-value = 0.018, one-way analysis of variance with Fisher’s least significant difference post-test for multiple comparisons.

bi-UKA, bi-unicompartmental knee arthroplasty; NKSS, New Knee Society Score; OKS, Oxford Knee Score; SD, standard deviation; TKA, total knee arthroplasty; VAS, visual analogue scale.

A sub-group analysis of OKS, NKSS, and pain VAS scores was conducted to compare the patients who perceived their allocation to be TKA (n = 6) or bi-UKA (n = 25) with those who remained unsure (n = 33) (Table IV). No significant differences were found in the OKS and NKSS scores between these groups preoperatively or at one year postoperatively. However, the data tended to favour better outcomes for those who believed that they were treated with a bi-UKA or remained unsure compared with those who believed that they were treated with a TKA. Following this trend, at one year postoperatively patients who believed that they were treated with a bi-UKA or remained unsure had significantly lower pain VAS scores (p < 0.002 and p < 0.018, respectively, one-way ANOVA with Fisher's least significant difference post-tests) compared with those who were treated with a TKA.

The tendency to guess incorrectly in favour of a bi-UKA may indicate a bias in favour of robotic surgery, or may simply reflect the fact that patients understood they had been enrolled into a robotic trial. Although we do not believe that this finding influenced the integrity of the trial, it highlights the importance of maintaining blinding in surgical trials.

## Discussion

The principle finding from this RCT was that there were no significant differences in the PROMS up to one year following surgery when comparing conventional TKA with robotic arm-assisted bi-UKA. Data on alignment and joint anatomy from this trial have already been reported by Banger et al^29^ who compared CT scans preoperatively and at three months following surgery in 38 TKAs and 32 bi-UKAs. The alteration in the anatomy of the joint after surgery was much less in patients undergoing a bi-UKA in all three planes in both the femur and tibia. Postoperative alignment was neutral in those who underwent TKA (179.5° (SD 3.2°)), while those who underwent bi-UKA had mild residual varus or valgus alignment (177.8° (SD 3.4°)).^29^ This element of the trial concluded that robotic-assisted, cruciate-sparing bi-UKA maintains the natural anatomy of the knee better in the coronal, sagittal, and axial planes and may therefore preserve normal joint kinematics, compared with a mechanically aligned TKA. Despite the clear differences in the preservation of joint anatomy and alignment following surgery, we found no association between this and improved clinical outcomes.

Confalonieri et al^20^ performed a matched cohort study which included 22 manually implanted bi-UKAs and 22 navigated TKAs. Similar to our findings, the postoperative HKAA alignment of TKAs (mean 179.4°) was significantly closer to neutral (p < 0.01), compared with bi-UKAs (mean 176.8°). However, unlike our findings, the bi-UKA group had significantly better function as measured by the Western Ontario and McMaster Universities Osteoarthritis Index function (p < 0.05) and stiffness (p < 0.01) at a minimum of four years follow-up (p < 0.05).^20^

It is not clear why we were unable to demonstrate any advantage in terms of PROMs with the bi-UKA approach in this study despite the maintenance of joint anatomy. The trial was powered for sagittal knee moment as the primary outcome measure and may have been underpowered for the assessment of PROMs. Outcomes following knee arthroplasty are multifactorial involving both patient and surgical factors. Although factors such as the preoperative psychological state, activity levels, and BMI were evenly distributed between the groups by the randomization process, the study was not large enough to be powered for the sub-group analysis that was carried out.

Although we have shown that the bi-UKA approach resurfaces the joint and maintains anatomy to a greater extent than TKA, the flat geometry of the Restoris tibial component does not mimic the curved anatomy of the surfaces of the native joint and their associated menisci. Perhaps if the concave geometry of the native joint could be replicated to a greater extent in compartmental arthroplasties of the knee in the future, particularly on the medial tibial side, this may further improve joint kinematics following bi-UKA, and show improved clinical outcomes. It is also difficult to recreate the convex geometry of the lateral tibia and the laxity in flexion seen in the native knee. Although flexion of the lateral tibial component helps to create more laxity in flexion, the bony anatomy of the lateral tibia has less slope than the medial side and laxity is created in flexion as the femur rolls back off the posterior aspect of the convex tibia.

We were satisfied to show equivalent outcomes between the surgical approaches and equivalent complications at up to one year following surgery. We acknowledge the increased operating time and additional costs associated with the procurement and maintenance of the robotic technology and the preoperative CT scans, disposables, and implants required to deliver a bi-UKA. Yet, as shown by Clement et al,^43^ the increased costs associated with robotic arm-assisted surgery for knee arthroplasty can be justified from health economic models.^43^ However, on the basis of our results, we are unable to recommend bi-UKA as a routine alternative treatment for patients with OA of the knee at present. We are also unable to identify which patients might benefit from this approach with the limited data that this study generated.

The accuracy of robotic arm-assisted technology, however, gives the opportunity of revisiting the way that arthroplasty of the knee is delivered and further studies, particularly with implants specifically designed for a multicompartment approach to resurface the knee, may deliver outcomes that surpass those currently seen with traditional techniques. Once improved outcomes can be demonstrated, any increased costs associated with the technique can be balanced against the clinical improvements.

The study had limitations. There were seven crossovers from bi-UKA to TKA, which potentially affected the independence of the randomization process. Due to this, intention-to-treat and per protocol analyses were carried out, which showed similar outcomes in both groups. We elected to present the per protocol analysis in this paper as most of the crossovers were due to random events linked to technical failures rather than true differences in the pathology being treated. In this cohort, there were three ACL-deficient patients in the TKA group and two in the bi-UKA group which were crossed over to TKA intraoperatively. A comparison of these patients to those with an intact ACL identified no significant differences between the groups during the study period. These patients were therefore included in the analysis.

A further limitation is that this is a secondary analysis of a trial powered for a primary outcome measure of biphasic gait. The numbers in the trial meant that there was insufficient power to determine significant differences between the groups for some of the outcome measures, particularly in subgroup analyses. Despite this, we believe that there is sufficient interest in the outcomes of bi-UKA for the early postoperative outcomes to be reported separately.

The results of our assessment of blinding underline the importance of maintaining blinding during follow-up. It is crucial to avoid potential bias towards new technologies from patients, researchers, surgeons, and other staff influencing the results of a study. All attempts were made to reduce this by maintaining identical pathways including a preoperative CT scan in both groups. However, the potential still exists when patients undergo surgery under regional anaesthesia rather than general anaesthesia or when research staff are urged to reveal the treatment allocation so that inadvertent unblinding occurs. Odgaard et al,^44^ in an RCT comparing patellofemoral arthroplasty (PFA) with TKA for patients with patellofemoral OA, incorporated an unblinding one year after surgery to reduce the pressure on unblinding before that time. In their study, at the one-year appointment patients were asked to guess their allocation and of 50 patients undergoing PFA, 19 and 18 guessed PFA and TKA, respectively, and of 50 patients undergoing TKA, the guesses were 13 and 25, respectively (p = 0.214); the missing answers represent patients who were unsure of their allocation. Our findings were remarkably similar and the failure to guess correctly which operation patients had undergone by those who thought they were ‘unblinded’ reassures us that this was unlikely to have influenced our results.

In conclusion, robotic arm-assisted, cruciate-sparing bi-UKA offers similar early clinical outcomes both in the immediate postoperative period and up to one year following surgery compared with a mechanically aligned TKA. In addition, subgroup analysis for preoperative psychological state, activity levels, and BMI showed similar outcomes between the two types of surgery. Superior outcomes were seen in patients who believed that they had undergone robotic-assisted surgery compared with those who believed that they had undergone TKA or were unsure of their allocation, particularly with VAS pain scores at one year (p < 0.05). Further work is required to identify which patients, such as younger, more active patients, might derive benefit from a cruciate-sparing bi-UKA.

**Take home message**

- Robotic arm-assisted bi-unicompartmental knee arthroplasty (bi-UKA) delivers similar clinical outcomes at all timepoints up to one year following surgery compared with a mechanically aligned total knee arthroplasty (TKA).

- Robotic arm-assisted bi-UKA has a similar safety profile to traditional mechanically aligned TKA surgery over the first postoperative year.
